# Current status and challenges for cell-cultured milk technology: a systematic review

**DOI:** 10.1186/s40104-024-01039-y

**Published:** 2024-06-08

**Authors:** Hyuk Cheol Kwon, Hyun Su Jung, Vahinika Kothuri, Sung Gu Han

**Affiliations:** Department of Food Science and Biotechnology of Animal Resources, Konkuk University, Seoul, Republic of Korea

**Keywords:** Cell culture system, Cell-cultured milk, Mammary epithelial cells, Precision fermentation

## Abstract

Cellular agriculture is an innovative technology for manufacturing sustainable agricultural products as an alternative to traditional agriculture. While most cellular agriculture is predominantly centered on the production of cultured meat, there is a growing demand for an understanding of the production techniques involved in dairy products within cellular agriculture. This review focuses on the current status of cellular agriculture in the dairy sector and technical challenges for cell-cultured milk production. Cellular agriculture technology in the dairy sector has been classified into fermentation-based and animal cell culture-based cellular agriculture. Currently, various companies synthesize milk components through precision fermentation technology. Nevertheless, several startup companies are pursuing animal cell-based technology, driven by public concerns regarding genetically modified organisms in precision fermentation technology. Hence, this review offers an up-to-date exploration of animal cell-based cellular agriculture to produce milk components, specifically emphasizing the structural, functional, and productive aspects of mammary epithelial cells, providing new information for industry and academia.

## Introduction

The dairy farming system has been directed toward enhancing efficiency of milk production through concentrated animal feeding operations, larger herds, advanced breeding technologies [1]. Over the past 80 years, milk yield in dairy farming has witnessed a 16.7-fold increase, from 53 million metric tons (Mt) in 1944 to 887 Mt in 2021 [2–4]. Furthermore, global milk production is forecasted to increase to 1,060 Mt by 2031 [3]. Genetic improvement has been a significant contributor to the increase in milk productivity. Specifically, three factors including transitioning from breeds such as Jersey and Guernsey to Holstein, widespread adoption of artificial insemination, and advancements in genetic evaluation procedures have played pivotal roles. These factors have collectively driven notable genetic changes in milk productivity [2].

Dairy intensification has been associated with adverse effects on the environment [4], animal welfare [5], human health [6], and rural livelihoods [1]. From an environmental standpoint, the intensification of dairy farming, encompassing enteric and manure storage, concentrated feed production, and farm crop cultivation, leads to greenhouse gas emissions, soil acidification, and eutrophication [7]. In addition, animals in dairy farms are raised in highly artificial environments to maximize milk yield, prompting concerns about animal welfare [8]. Nitrate contamination of soil, aquifers, and rivers through the accumulation of cattle urine in dairy farming is another major concern for human health, as exposure to such contaminated water is associated with colorectal cancer [9]. Thus, dairy farming and industries are focusing on producing milk and milk products in a sustainable rather than traditional manner [10].

Cellular agriculture is receiving attention as a new sustainable technology for agricultural food production that can incrementally positively affect the environment and society [11]. Cellular agriculture holds considerable promise over traditional agriculture, offering potential benefits in terms of environmental sustainability, economic value, enhanced animal welfare, and improved human health and well-being [12]. Several companies across the globe are focusing on the production of cellular agricultural products, such as cultured meat and cell-cultured milk, based on cellular agriculture technology [13]. Nevertheless, this technology has been predominantly applied for production of cultured meat [11, 14, 15]. Therefore, comprehensive studies are required to understand the production of sustainable cell-cultured milk. This review aimed to comprehensively identify the current status of cellular agriculture in the dairy sector and to understand the fundamental knowledge and challenges associated with cell culture-based dairy technology.

## Cellular agriculture in the dairy sector

**Fig. 1:**
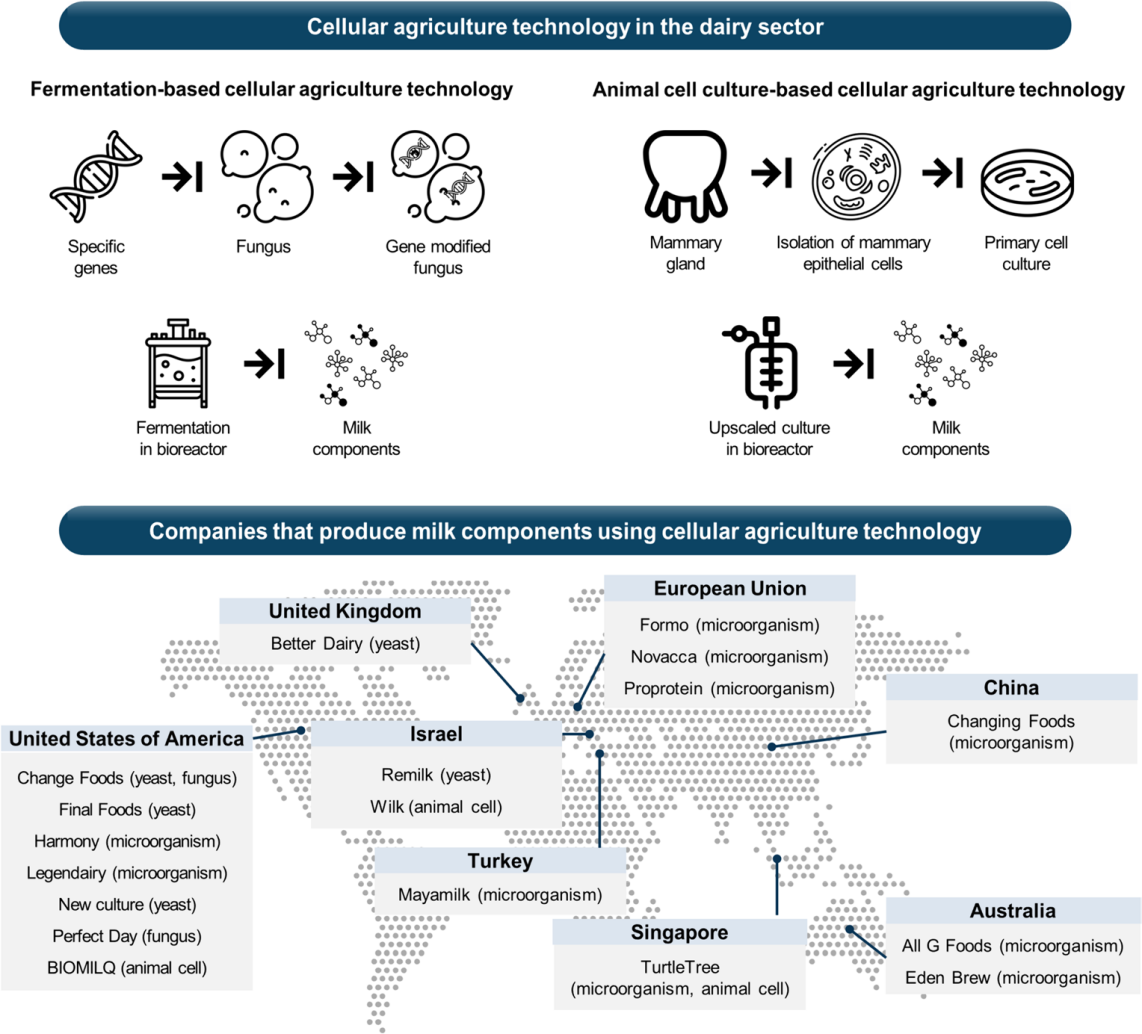
Cellular agriculture technology in the dairy sector and global startup companies to produce milk components. Various global companies are trying to synthesize milk components through fermentation- and animal cell culture-based technology

Cellular agriculture is a sustainable manufacturing technology that produces products such as meat components, milk components, and egg proteins using a cell culture system [16]. Various companies are currently trying to produce cell-cultured milk components using fermentation-based and animal cell culture-based technology (Fig. 1) [17]. However, prohibitive cost involved in the research, development, and production remain major obstacles [18]. Therefore, this section covers the techno-economic cost and technical state in current cell culture-based dairy field.

### Techno-economic analysis

To assess the economic viability of cell-cultured milk components, a techno-economic analysis was conducted to estimate the cost-effectiveness of cell cultured milk components compared to whole milk. In the United States, a liter of whole milk typically costs around $1.00 USD [19]. One liter of milk contains about 26 g of casein (13 g of α-casein, 9.3 g of β-casein, and 3.3 g of κ-casein) and 40 g of fats [20]. Our previous research showed that MAC-T cells grown in a progesterone (P4)-based differentiation media were able to produce some key milk components [21]. Specifically, these cells synthesized 0.515 g of α-casein and 12.19 g of triglycerides per liter of media. However, it is important to consider that this media itself costs about $175 USD per liter [21]. Based on this cell-cultured milk technology, producing the same amounts of α-casein and triglycerides found in a liter of whole milk would be very expensive. The production for α-casein and triglycerides would likely cost around $4,417 USD and $574 USD, respectively. It is important to remember that this only considers the cell culture media. The actual production cost would be much higher because it excludes costs for labor, production facilities, separation and purifying the desired milk components, and other miscellaneous expenses. As a result, these projections indicate that the significant production expenses pose a significant obstacle in contemporary cellular agriculture. Therefore, it is important to develop cost-efficient methodologies to facilitate large-scale industrial production [18]. The primary focus of technical advancement involves enhancing cell lines, developing low-cost yet high-performance medium, optimizing bioreactors for efficiency, and refining down-stream processing methods for cost-effectiveness. These efforts are essential for realizing economically viable production of cell-cultured milk components.

### Fermentation-based cellular agriculture

Fermentation-based cellular agriculture employs synthetic biology and genetic engineering to introduce specific genes into the DNA backbone of bacteria, yeast, or algae, to produce desired products [22]. Based on precision fermentation technology, various companies have developed and commercialized milk components, such as casein, whey protein, and lactoferrin (Table 1). However, most companies have chosen not to disclose details regarding the microorganisms or the techniques they utilized.

**Table 1:** Companies that produce milk components using fermentation-based technology in cellular agriculture

Company	Products	Microorganisms	Location	References
AII G Foods	Milk proteins	Undisclosed	Sydney, Australia	[22]
Better Dairy	Casein	Yeast	London, United Kingdom	[22, 23]
Change Foods	Casein	Bacteria, yeast, filamentous fungi	California, United States of America	[22, 23]
Changing Biotech	Undisclosed protein	Undisclosed	Shanghai, China	[23]
Eden Brew	Milk proteins	Undisclosed	Sydney, Australia	[22]
Final Foods	Whey proteins	Yeast	California, United States of America	[22]
Formo	Casein and whey protein	Undisclosed	Berline, Germany	[23]
Harmony	Human milk proteins	Undisclosed	Massachusetts, United States of America	[22]
Legendairy	Milk proteins	Undisclosed	Texas, United States of America	[22]
Mayamilk	Milk proteins	Undisclosed	Izmir, Turkey	[22]
New Culture	Casein	Yeast	California, United States of America	[23, 24]
Novacca	Milk proteins	Undisclosed	Denmark	[22]
Perfect Day	β-Lactoglobulin	Fungus	California, United States of America	[22–24]
Proprotein	Casein	Undisclosed	Tallinn, Estonia	[22]
Remilk	Casein and β-lactoglobulin	Yeast	Rehovot, Israel	[23, 24]
TurtleTree	Lactoferrin	Undisclosed	Singapore	[24]

Fungi are known as the most suitable microbial hosts for precision fermentation because of their strong environmental adaptability [25]. From the metabolic engineering perspective, compared to bacteria, fungi possess better eukaryotic properties that allow them to express heterologous eukaryotic proteins, correcting protein folding and post-translation modifications [26, 27]. The filamentous fungus *Trichoderma reesei* is commonly utilized to synthesize recombinant food components because of its high protein productivity (up to 100 g/L); moreover, it is generally regarded as safe [22, 25]. Indeed, a prior study documented that *T. reesei*, employing precision fermentation technology, can produce β-lactoglobulin (BLG) at a level of 1 g/L, with the structural and functional properties of the recombinant BLG being consistent with bovine BLG [28].

Precision fermentation has enabled the production of sustainable milk components, an emerging food trend in the fourth industrial revolution of the food industry [29]. However, the commercialization of genetically modified organisms (GMOs) used in precision fermentation has raised public concerns about food safety [30]. Accordingly, the production of food components using GMOs requires careful regulation, thorough safety evaluations, and consideration of consumer concerns [22]. Therefore, although precision fermentation as an innovative technology is anticipated to reduce the reliance on traditional dairy farming, overcoming GMO concerns remains a major challenge for fermentation-based cellular agriculture.

### Animal cell culture-based cellular agriculture

Animal cell and tissue culture-based cellular agriculture involves tissue engineering to produce functional tissues using minimal cells or cell lines obtained from living animals [16]. Recent biotech startups have emerged, securing funds to pioneer the development of cell-cultured milk production (Table 2) [31]. As current animal cell culture-based cellular agriculture has technical difficulties in synthesizing whole milk, they mainly aim to produce a single component of milk using mammary epithelial cells (ECs) [32].

**Table 2:** Companies that try to produce milk components using animal cell culture-based technology in cellular agriculture

Company	Products	Animal cells	Location	References
BIOMILQ	Bovine and human milk components	Mammary epithelial cells	North Carolina, United States of America	[33]
Turtle Tree	Goat and human milk component	Mammary epithelial cells	Singapore	[33]
Wilk	Bovine and human milk components	Mammary epithelial cells	Rehovot, Israel	[23, 32]

Milk components, such as casein, whey protein, and triglycerides, are primarily synthesized and secreted by ECs of the mammary gland [34, 35]. Thus, the primary step in the in vitro production of cell-cultured milk components is to obtain ECs. Companies such as BIOMILQ and Wilk isolate ECs from the milk-secreting parenchymal tissue of the mammary gland. In contrast, Turtle Tree isolates mesenchymal stem cells from mammary tissues, adipose tissues, and the umbilical cord, subsequently inducing differentiation into ECs [23, 32]. Despite the focus of the mentioned startup companies on the production of cell culture-based milk, technology related to animal cell culture for producing milk components is still in its early stages. Consequently, novel strategies are essential to surmount the technical barriers of animal cell culture-based cellular agriculture, necessitating a deeper understanding of milk biosynthesis in the mammary gland.

## Current knowledge and technical challenges for producing cell-cultured milk

**Fig. 2:**
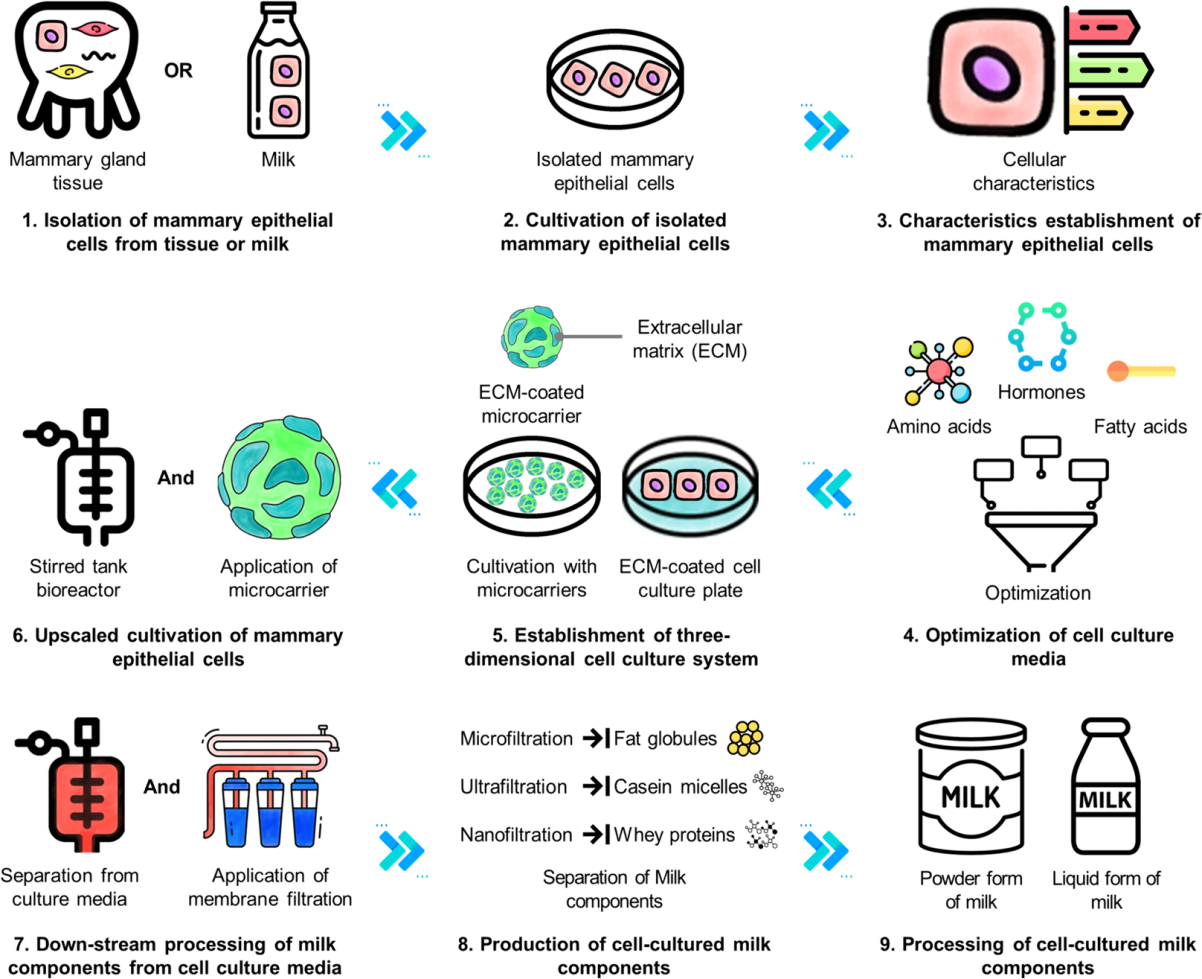
Principal processes and technical challenges for producing cell-cultured milk using animal cell culture-based technology. The production of cell-cultured milk, found on the cultivation of mammary epithelial cells (ECs), is through a series of sequential processes as follows: (1) isolation of mammary ECs from parenchymal tissues or milk, (2) cultivation of isolated mammary ECs for the establishment of a cell line, (3) evaluation of cellular characteristics, (4) optimization of cell culture media, (5) establishment of three-dimensional cell culture system using extracellular matrix, (6) upscale of cell cultivation using stirred tank bioreactor and microcarrier, (7) down-stream processing of cell culture media, (8) production of cell-cultured milk components, and (9) processing of cell-cultured milk components. The major technical challenges for the production of cell-cultured milk are resourcing the cell line (1–3), optimizing the cell culture media (4), establishing the cell culture system (5–6), and separating milk components (7–8). Comprehensive and detailed technical challenges for cell-cultured milk production are discussed in this review

The mammary gland itself is a complicated bioreactor comprised of alveolar structure including various cell types. Technical challenges for producing cell-cultured milk include replicating the structure of milk-secreting mammary glands and reconstructing it within an in vitro environment. Cell-cultured milk can be produced through the intricate processes such as the structural interaction of cells and the regulation of milk synthesis-related hormones while cell-cultured meat is generally produced by culturing muscle cells or adipocytes [36]. Therefore, a detailed process of the milk synthesis and secretion in the mammary gland to produce cell-cultured milk would be described in this section. The short-term objective is to produce individual milk components using a two-dimensional (2D) culture of mammary cells, while the ultimate long-term goal is to achieve the production of whole milk through the three-dimensional (3D) culture of mammary glands [15]. To accomplish these objectives, the primary technical challenges will involve ensuring the sustainable resourcing of mammary cells, optimizing cell culture media, establishing a robust cell culture system, and down-stream processing of cell-cultured milk components (Fig. 2) [32].

### Understanding functional and structural features of the mammary gland

**Fig. 3:**
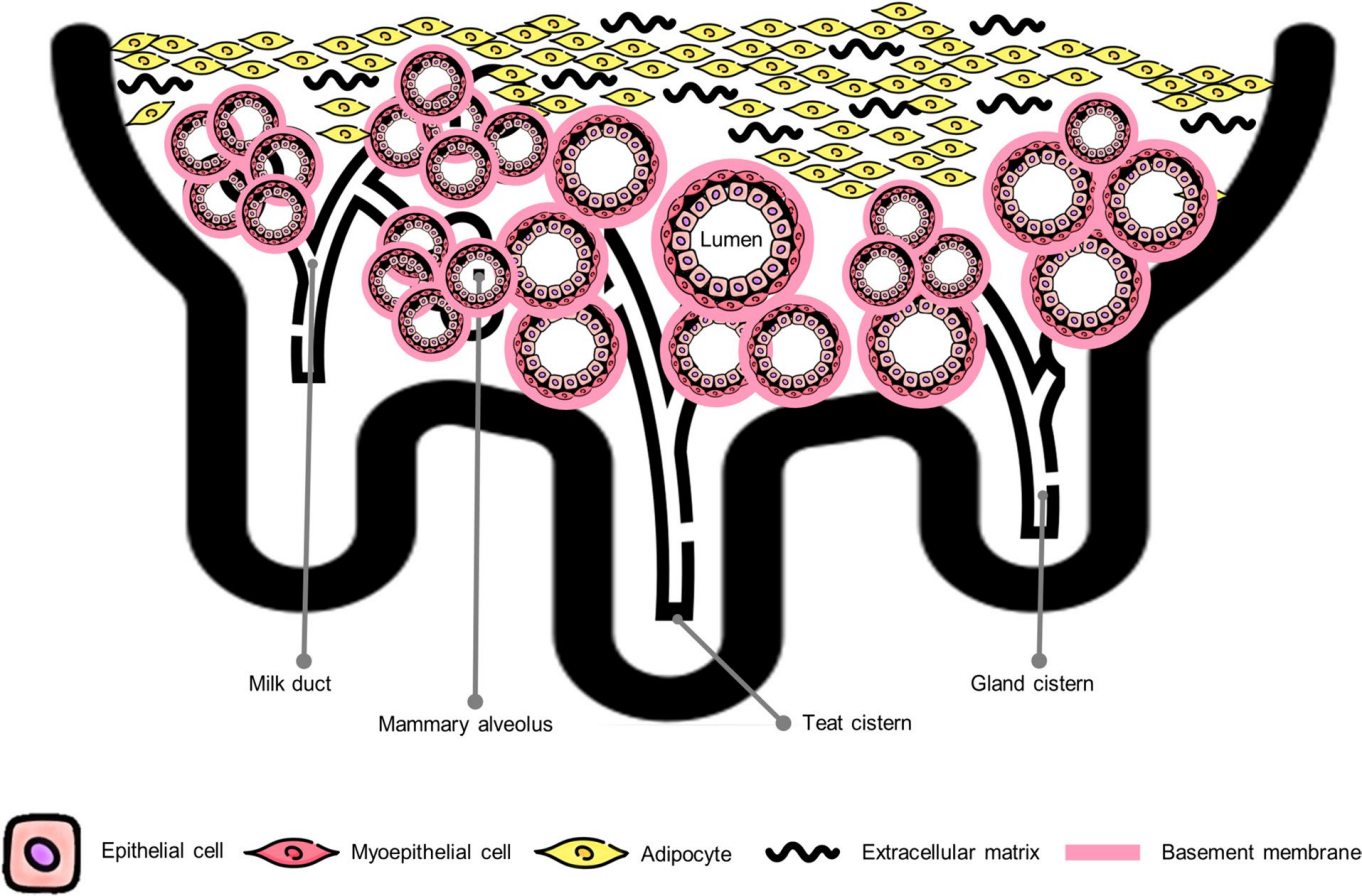
Internal structure of mammary gland and mammary alveolus. The mammary gland is composed of lobes that comprise lobules containing 150–220 alveoli. Mammary alveoli are fundamental constituents that produce milk components. The mammary alveolus consists of parenchymal and stromal compartments based on the basement membrane. The parenchyma is constructed of inner milk secretory epithelial cells that surround a central lumen and outer myoepithelial cells that attach to the base of the mammary epithelium. In addition, the stroma is constituted of adipocytes and extracellular matrix

The mammary gland (breast) is a distinctive organ exclusive to mammals, characterized by an anatomical structure designed for the secretion of milk to nourish a newborn (Fig. 3) [37]. Herein, mammary alveolus is a fundamental constituent of mature mammary glands for milk production. Alveolar parenchyma comprises inner milk secretory ECs that surround the lumen, outer myoepithelial cells (MCs) that attach to the basal mammary epithelium, and the basement membrane (BM) that contacts the MCs. The stromal compartment comprises various stromal cells, including fibroblasts (FBs), adipocytes, endothelial cells, and extracellular matrix (ECM) [35, 38]. Thus, milk components are structurally synthesized by ECs, contracted by MCs, and secreted into the lumen [39].

The functional capabilities of the mammary gland for milk synthesis and secretion occur primarily during physical development [40]. The mammary gland develops throughout the four growth stages (i.e., pre-puberty, post-puberty, pregnancy, and lactation), experiencing repeated apoptosis and growth in response to pregnancy cycles, parturition, lactation, and involution [40, 41]. In particular, the mature functional development of the mammary gland, which directly enables the synthesis and secretion of milk, occurs primarily during pregnancy and lactation [42], and is primarily regulated by the reproductive and metabolic hormones. Among the various hormones, prolactin (PRL) and P4 directly induce the alveologenesis and secretory differentiation through receptor activator of nuclear factor kappa-B ligand during pregnancy. In addition, 17β-estradiol (E2), cortisol (CORT), insulin (INS), and growth hormones support this development of mammary gland. After that, a decrease of P4 concentration in the presence of PRL, CORT, and INS triggers the secretory activation and the onset of milk production for the transition to lactation [43]. These features of the mammary gland are essential for the synthesis of milk components and are tightly regulated through the coordinated action of hormones within mammary cells.

Considering the structural and functional properties of the mammary gland as summarized above, the activation of mammary ECs or secretory differentiation through hormonal regulation are essential for producing cell-cultured milk components. To initiate this process in the laboratory, the first step involves isolating milk-secreting ECs and establishing their cellular characteristics.

### Resourcing of mammary epithelial cells

ECs can be isolated from the mammary gland tissues or milk of animals and humans, depending on the type of milk desired for production (Table 3). Isolating ECs from tissue is typically used in current research field due to its technical ease to apply. However, as the ECs can be obtained from the parenchymal tissue of the mammary gland after biopsy or slaughter, the procedures are uneconomical, time-consuming, and inconvenient. Furthermore, as mammary gland tissue comprises various cell populations such as ECs, MCs, FBs, adipocytes, and ECM [35], disassociating mammary gland tissue is essential to isolate ECs. Diverse enzymes such as collagenase, hyaluronidase, and trypsin have been used to isolate and purify ECs. In particular, trypsin has been widely used to remove FBs from the mammary gland tissue. However, according to a recent study, the combination of collagenase type 1 and hyaluronidase more effectively isolated ECs with better preservation of the physiological properties than trypsin [44]. Therefore, optimization of dissociation by applying multiple enzyme combinations would improve the physiological properties of isolated ECs, contributing to cell line resourcing.

**Table 3:** Dissociation and sorting methods for the isolated mammary epithelial cells from the mammary gland and milk

Species	Sources	Dissociation enzymes	Sorting methods	Markers	References
Buffalo	Mammary gland	Collagenase, hyaluronidase, and trypsin/EDTA	Selective trypsinization	CK18, vimentin	[45]
Caprine	Mammary gland	Collagenase type 1	Collagen digestion	CK18, CK19, vimentin, α-SMA	[46]
Dairy cow	Mammary gland	Trypsin/EDTA	Selective trypsinization	CK18, vimentin	[47]
Dairy cow	Mammary gland	Trypsin/EDTA	Selective trypsinization	CK18	[48]
Dairy cow	Mammary gland	Trypsin/EDTA	Selective trypsinization	CK18	[49]
Dairy cow	Mammary gland	Trypsin/EDTA	Selective trypsinization	Pan-CK	[50]
Dairy cow	Milk	Not applicable	Centrifuge 1,850 × *g*, 10 min	Pan-CK	[51]
Dairy cow	Milk	Not applicable	Centrifuge 1,850 ×* g*, 10 min	CK8, Pan-CK	[52, 53]
Goat	Mammary gland	Collagenase type 1, Trypsin/EDTA	Selective trypsinization	CK18	[54]
Human	Mammary gland	Collagenase, hyaluronidase, Accumax	Collagen digestion	CK8, CK14, CK18	[55]
Porcine	Mammary gland	Collagenase A, hyaluronidase	Collagen digestion	CK18, vimentin	[56]
Yak	Mammary gland	Trypsin/EDTA	Selective trypsinization	CK8, CK18, vimentin	[57]

*CK* Cytokeratin, *EDTA* Ethylene-diamine-tetraacetic acid, *α-SMA* α-Smooth muscle actin

Recently, isolating ECs from milk is receiving attention as a novel strategy because it has several advantages, including non-invasiveness, repeatability, and less contamination by FBs. Notably, the cytoskeletal characteristics and milk productivity of primary bovine ECs extracted from milk were comparable to those of cells isolated from tissue [58]. In addition, human breast milk was a rich source of heterogeneous cell types such as milk-secreting ECs, MCs, progenitor cells, and multipotent mesenchymal stem cells [59, 60]. Therefore, isolating ECs from milk can be another effective alternative method for sustainable resourcing of ECs [52]. However, further study is required to demonstrate the suitability as an alternative to the tissue culture regarding gene expression and cellular functionality.

The cytoskeleton plays a crucial role in cellular integrity, structure, and function and expresses specific cytoskeletal proteins depending on the cell type [32]. Milk-producing ECs specifically express cytokeratin (CK) 8 [61]. ECs, MCs, and FBs selectively express CK18/19, α-smooth muscle actin (α-SMA), and vimentin as specific markers, respectively [62]. Accordingly, various cytoskeletal protein markers, including CK8, CK18, CK19, vimentin, and α-SMA, can be utilized to distinguish mammary cell line types. Taken together, isolating ECs from milk and evaluating reliable biomarker would contribute to the stable resourcing of the mammary cells.

### Optimization of cell culture media for mammary epithelial cells

Optimal cultivation conditions for ECs can be established by imitating the in vivo circulatory system and physiological environment of the mammary alveolus. As all of the precursors for milk production are supplied from the blood [63], it plays an important role in providing hormones and nutrients for the growth, development, and lactation of the mammary gland [64]. Therefore, the most fundamental factor for the production of cell-cultured milk is to optimize the growth and differentiation media of ECs based on the levels of constituents in the blood during the development and lactation of the mammary gland [65].

Cell culture media are composed of a basal medium (comprising amino acids, vitamins, inorganic salts, glucose, among others), serum or serum alternatives (source of growth factors, hormones, and attachment factors), and several supplements [66]. Generally, Dulbecco’s Modified Eagle’s Medium/Nutrient Mixture F-12 Ham (DMEM/F12) with the addition of 10% fetal bovine serum (FBS) is used for cultivating ECs. In addition, antibiotics such as penicillin, streptomycin, gentamicin, and amphotericin B are added for aseptic cell culture. Therefore, the most fundamental growth media for the cultivation of ECs consist of DMEM/F12, 10% FBS, 1% penicillin/streptomycin, and 5 µg/mL amphotericin B (Table 4).

**Table 4:** Culture conditions for the isolated mammary epithelial cells from mammary gland and milk

Species	Basal media	Serum	Antibiotics	Hormones	Other additives	References
Buffalo	DMEM/F12	FBS (10%)	Penicillin (100 U/mL), streptomycin (5 µg/mL), amphotericin (50 ng/mL)	INS (5 µg/mL), CORT (1 µg/mL), EGF (10 ng/mL), PRL (5 µg/mL)	Transferrin (1 µg/mL)	[45]
Caprine	DMEM/F12	FBS (10%)	Penicillin (100 U/mL), streptomycin (100 µg/mL)	INS (10 µg/mL), CORT (5 µg/mL)	Sodium bicarbonate (2.2 mg/mL), sodium acetate (5 mmol/L), holo-transferrin (5 µg/mL), ethanolamine (0.5 mmol/L),	[46]
Dairy cow	DMEM/F12	FBS (10%)		INS (5 µg/mL), P4 (5 µg/mL), CORT (1 µmol/L), EGF (10 ng/mL), E2 (5 µg/mL)	Transferrin (5 µg/mL)	[47]
Dairy cow	DMEM/F12	FCS (10%)	Penicillin (1%), streptomycin (1%)	INS (5 µg/mL), CORT (1 µg/mL), EGF (10 ng/mL), PRL (5 µg/mL)	Transferrin (5 µg/mL), glutamine (1%)	[48]
Dairy cow	DMEM/F12	FBS (10%)	Penicillin/streptomycin (1%)	EGF (1%)		[49]
Dairy cow	DMEM/F12	FBS (20%)	Penicillin/streptomycin (50 IU/mL), amphotericin B (2.5 µg/mL)	INS (1 µg/mL)		[50]
Dairy cow	DMEM/F12, INS (5 mg/mL)	FCS (10%)	Penicillin/streptomycin (100 µg/mL), gentamycin (100 µg/mL), amphotericin B (5 µg/mL)		Transferrin (5 mg/mL), sodium selenite (5 µg/mL)	[51]
Dairy cow	DMEM/F12	FBS (10%)	Amphotericin B (1.76 µg/mL)	INS (10 µg/mL), CORT (1 µg/mL),	Transferrin (5 µg/mL), sodium selenite (5 ng/mL)	[52, 53]
Goat	DMEM/F12	FBS (10%)	Penicillin/streptomycin (100 IU/mL)	ITS (5 ng/mL), IGF-1 (10 ng/mL), EGF (10 ng/mL)		[54]
Human	DMEM/F12	FBS (5%)		INS (5 µg/mL), CORT (0.4 µg/mL), EGF (10 ng/mL)	Cholera toxin (8.4 ng/mL), adenine (24 µg/mL), Y-27632 (10 µmol/L)	[55]
Porcine	DMEM/F12	FBS (10%)		INS (0.5 µg/mL), CORT (1 µg/mL), PRL (0–2 µg/mL)		[56]
Yak	DMEM/F12	FBS (10%)	Penicillin (100 IU/mL), streptomycin (5 µg/mL)	ITS (5 µg/ML), EGF (5 ng/mL), CORT (1 µg/mL), P4 (5 µg/mL)		[57]

*CORT* Cortisol, *DMEM/F12* Dulbecco’s Modified Eagle’s Medium/F-12 Nutrient Mixture Ham, *E2* 17β-estradiol, *EGF* Epidermal growth factor, *FBS* Fetal bovine serum, *FCS* Fetal calf serum, *IGF-1* Insulin growth factor-1, *INS* Insulin, *ITS* Insulin-transferrin-selenium, *P4* Progesterone, *PRL* Prolactin

Amino acids, fatty acids, glucose, vitamins, and minerals are key nutrients for the structural development of and milk component biosynthesis by ECs [67]. Reproductive and metabolic hormones such as PRL, P4, E2, CORT, and INS are also essential for the proliferation and differentiation of ECs [43]. Therefore, amino acids and hormones can promote the proliferation and differentiation of ECs [43, 67, 68]. The increase in the number of ECs through proliferation and differentiation enhanced the milk productivity in the development and lactation of mammary gland [69]. In addition, milk fat composed of triglycerides (98%), diglycerides (about 2%), cholesterol (less than 0.5%), phospholipids (about 1%), and free fatty acids (about 0.1%), are mainly biosynthesized by ECs from more than 400 different fatty acids [70]. The most abundant fatty acids in milk consist of long-chain fatty acids in the order of palmitic acid (C 16:0), oleic acid (18:1), stearic acid (18:0), and myristic acid (14:0) [71]. These long-chain (C18 and some C16) fatty acids are derived from the blood plasma lipid originating from the diet, while medium- and short-chain fatty acids are synthesized through de novo synthesis in ECs [72, 73]. Therefore, various additives, including hormones, amino acids, and fatty acids, can be supplemented to the cell culture media to promote the proliferation and differentiation of ECs, thereby enabling the production of cell-cultured milk components [74]. Finally, the optimal proliferation and differentiation media needs to be established based on the concentration of hormones, amino acids, and fatty acids in blood plasma during pregnancy and lactation (Table 5).

**Table 5:** Concentration of amino acids, hormones, and fatty acids in the bovine blood plasma

Categories	Bioactive compounds	Concentration	References
Essential amino acids (µg/g of amino acids)	Arginine	13.24−33	[75, 76]
	Isoleucine	10.75−33.5	
	Histidine	4.65−42	
	Leucine	15.20−93.4	
	Lysine	9.21−74.7	
	Methionine	3.28−8.6	
	Phenylalanine	14.70−51.6	
	Threonine	7.03−66	
	Tryptophan	11.8−19.40	
	Valine	22.26−67.3	
Hormones (ng/mL in blood plasma)	INS	0.35 (puberty), 0.416−0.625 (pregnancy−lactation), 0.25−0.5 (5 d before the onset of lactation)	[77, 78]
	CORT	9 (pregnancy), 3−5 (lactation), 5 (5 d before the onset of lactation)	[79–81]
	P4	0.5−3.5 (lactation), 4.5−6.5 (5 d before the onset of lactation)	[81, 82]
	E1	3.5 (pregnancy), 0.05 (lactation)	[83]
	E2	0.55 (pregnancy), 0.025 (lactation), 0.5−0.8 (5 d before the onset of lactation)	[83]
	PRL	50 (5 d before the onset of lactation)	[78, 79, 84]
Fatty acids (µg/g of fatty acids)	Myristic acid (14:0)	7.7−10.2	[85–87]
	Palmitic acid (16:0)	120−209	[85–87]
	Palmitoleic acid (16:1)	25.4−58	[85, 86]
	Stearic acid (18:0)	154.6−188	[85–87]
	Oleic acid (18:1)	86.5−149.6	[85–87]
	Linoleic acid (18: 2n-6)	280.2−376	[85–87]
	Docosahexaenoic acid (n-3)	10.3−34.2	[85, 86]
	Arachidonic acid (n-6)	414−421	[85, 86]
	SFA	402−405	[85, 86]
	MUFA	141−174	[85, 86]
	PUFA	425−456	[85, 86]

*CORT* Cortisol, *E1* Estrone, *E2* 17β-estradiol, *INS* Insulin, *MUFA* Monounsaturated fatty acid, *P4* Progesterone, *PRL* Prolactin, *PUFA* Polyunsaturated fatty acid, *SFA* Saturated fatty acid

### Establishment of a cell culture system for mammary epithelial cells

ECs have typically been cultured using the 2D cell culture method to study the function of the mammary gland [49, 50]. Two-dimensional cultivation has the experimental advantage of promoting homogenous growth and proliferation of cells by supplying a consistent amount of nutrients and growth factors from cell culture media [88]. However, as 2D cell culture cannot completely mimic the structural shape of the tissues observed in the mammary gland, the bioactivities of the cells appear considerably different compared to those of tissues [89]. Furthermore, it has been reported that 2D-based cell culture is manual- and labor-intensive, demanding a significant amount of space and incurring a high manufacturing cost [90]. Consequently, 2D cell culture raises several strategic problems from the perspective of structural and productive cultivation of ECs for producing cell-cultured milk.

A 3D cell culture system is a potential approach for more effective production of cell-cultured milk. The first goal for 3D cell culture is to precisely mimic the structural and functional formation of mammary gland tissue. From the structural and functional perspectives of mammary alveolus, ECs are in contact with the thin and dense layers of a specialized ECM, termed the BM [89]. The BM, composed of a polymeric network of proteins, including laminins, collagen IV, heparin sulfate proteoglycan, and nidogen, has been reported to interact with ECs in the proliferation, differentiation, and metabolism processes [91–93]. Indeed, the culture of ECs with 3D collagen gels resulted in the maintenance of differentiation and synthesis of milk proteins, suggesting that ECM plays a key regulatory role in 3D cell culture [94, 95]. Therefore, the application of BM proteins is required for establishing a 3D cell culture of ECs.

Spheroids and scaffolds represent various 3D cell culture strategies. Additionally, microcarrier technology has emerged as suitable tool to apply BM proteins [96, 97]. Microcarriers are support matrices 100−300 μm diameter that enable the cultivation of anchorage-dependent adherent cells in a bioreactor system [98]. Several microcarriers are commercially synthesized using various materials, including glass, dextran, polystyrene, cellulose, collagen, gelatin, collagen, alginate, and chitosan [99]. These microcarriers can be coated with ECM proteins, such as laminin, collagen, and fibronectin, for the efficient adhesion of the cells. ECM proteins provide many RGD tripeptide (arginine-glycine-aspartate) motifs that can specifically bind to cell surface receptors [100]. Therefore, ECM proteins can enhance the cell attachment of microcarriers along with a high surface-to-volume ratio [101]. Indeed, a culture of 3 g/L Cytodex 1 (190 μm) and 3 (175 μm) microcarrier provides a surface area of 8.1–13.2 × 10^3^ cm^2^ in 1L, which is equivalent to 108–176 of 75 cm^2^ cell culture flasks [101]. Various types of microcarriers have been mainly applied to cultivate human mesenchymal and pluripotent stem cells for cell therapy in clinical trials [101–103]. However, most studies related to ECs have primarily utilized microcarriers to establish 3D in vitro breast tumor models [104, 105]. Only one study reported the optimal cell adhesion, growth, and differentiation conditions on collagen-coated microcarriers (Cytodex 3) using bovine mammary epithelial cell line MAC-T [106]. Therefore, the optimization and application of ECs to ECM-coated microcarriers are required to overcome the structural and productive limitations of synthesizing cell-cultured milk.

To facilitate the upscale production of ECs, it is imperative to introduce a culture system that is more space-, labor-, and cost-efficient. A bioreactor stands out as a promising culture system for the large-scale cultivation of ECs [107]. Bioreactors have been extensively used for the industrial large-scale cultivation of mammalian cells under a controlled microenvironment [108]. Therefore, several bioreactor systems (stirred tank, wave, rotating wall, hollow fiber, and packed-bed), primarily developed for cultivating conventional mammalian cells, can potentially apply to the cultivation and scale-up of ECs [109]. Among the various bioreactor systems, a stirred tank bioreactor has many advantages for the commercial scale production, including ease of design, scale-up, in situ monitoring, and operation in different batch. While this bioreactor does have limitations in meeting the oxygen demand of large volumes (such as high-density cell cultures) and controlling excessive shear stress caused by the impeller, these challenges can be addressed through process optimization strategies. [110]. Considering the structural and functional characteristics of ECs and the application of microcarriers, a stirred tank bioreactor would be one of the most applicable systems for cell-cultured milk production. Stirred tank bioreactor is one of the most conventional bioreactors, which consist of a tank equipped with an impeller for efficient mixing and suspension [110]. The impeller, a core component of a stirred tank bioreactor, controls the culture environment, including pH, dissolved oxygen, temperature, nutrients, and metabolites through agitation [109, 111]. These bioreactors are simple and easy to monitor and control for large-scale cell cultivation [112]. Indeed, mammary epithelial stem cells inoculated at a 7.5 × 10^4^ cells/mL in 125, 500, and 1,000 mL of stirred bioreactors resulted in the expansion of cell density of 3.38, 3.76, and 4.21 × 10^5^ cells/mL, respectively, with the formation of aggregates (mammospheres) [113]. Moreover, stirred culture systems have the advantage of easy application of microcarrier technology that enhances the productivity of cells and their derivatives through an increase in the high surface-to-volume ratio [102]. Comprehensively, applying an ECM-coated microcarrier in a stirred tank bioreactor would be the most suitable cell culture system for the upscaled production of cell-cultured milk.

### Down-stream processing of milk components from cell culture media

ECs cultured in a cell culture system secrete milk proteins and fat globules into the cell culture media. Concurrently, various cell culture additives including serum, hormones, and antibiotics used for the cellular proliferation and differentiation are contained in the cell culture media. However, only milk components should be separated and purified from the cell culture media. In these perspectives, membrane-based techniques can be effectively employed for isolating, purifying, and processing the milk components from the cell culture media.

Pressure-driven membrane separation process technology has widely been applied to produce high-value added dairy components in the dairy industry and to remove the hormones and antibiotics in the wastewater treatment industry [114, 115]. Membrane separation processes are classified as reverse osmosis (< 1 nm), nanofiltration (1–10 nm), ultrafiltration (10–100 nm), and microfiltration (100–10,000 nm) depending on the membrane pore size and molecular weight cutoff [114, 115]. One liter of milk contains 26 g casein micelles, 7 g whey proteins, and 40 g fat globules with sizes of 20–300 nm (average 110 nm), 3–6 nm, and 100–15,000 nm (average 3,400 nm), respectively [20, 116, 117]. Accordingly, fat globules, casein micelles, and whey proteins in the cell culture media can be separated using microfiltration, ultrafiltration, and nanofiltration [117]. In addition, since hormones and antibiotics have molecular weight of average 0.25–0.5 kDa, nanofiltration and reverse osmosis can be used for the removal [118, 119]. In detail, microfiltration, with a 1,400 nm pore size, is a standard method for separating fat globules and bacteria [120]. Microfiltration, with a 100–200 nm pore size, is employed to separate casein micelles from whey protein. Ultrafiltration and nanofiltration, featuring pore sizes of 1–100 nm and 2 nm, respectively, are used to concentrate whey protein [121]. Additionally, nanofiltration and reverse osmosis, which have a molecular weight cut off of 0.3–1 kDa and 0.1 kDa, respectively, are applied for removing the various types of hormones and antibiotics (Table 6) [114, 115].

**Table 6:** Membrane types based on milk components in membrane separation process technology

Membrane type	Pore size, nm	Molecular weight cut off (pressure)	Separation component (Size distribution, nm)	Reference
Microfiltration	100–10,000 100–200	> 200 kDa (low, below 2 bar, 0.2 Mpa)	Fat globules (100–15,000) Casein micelles (20–300)	[117–119, 122–124]
Ultrafiltration	1–100	1–200 kDa (medium, 1–10 bar, 0.1–1 Mpa)	Casein micelles (20–300) Whey proteins (3–6)	
Nanofiltration	1–10	0.3–1 kDa (medium to high, 5–40 bar, 0.5–4 MPa)	Whey proteins (3–6) Hormones and antibiotics (0.25–0.5 kDa)	
Reverse osmosis	< 1	0.1 kDa (high, 10–100 bar, 1–10 MPa)	Lactose and water (0.35 kDa) Hormones and antibiotics (0.25–0.5 kDa)	

Two types of tubular ceramic membranes (TCMs) and spiral-wound membranes (SWMs) are typically applied in the separation of milk components using microfiltration, ultrafiltration, and nanofiltration [117]. TCM is widely used for membranes because of its narrow pore distribution, high hydraulic performance, and high thermal stability [125, 126]. However, compared with SWM, TCM has high transmembrane pressure (∆p_TM_), which increases the flux value (L/m^2^·h) and membrane fouling, resulting in high energy consumption and low separation efficiency for milk protein fractionation [127]. Thus, recent studies have focused on optimizing the efficiency of milk protein separation using SWM to improve membrane fouling [128, 129]. A 0.3-µm pore size SWM can achieve a whey protein separation ratio of up to 97% from skim milk, surpassing the 95% ratio achieved with the 0.1-µm pore size TCM [128]. Furthermore, although pore size did not affect the flux value in 0.1- and 0.2-µm pore size SWM, the 0.1-µm pore size SWM was more suitable for milk protein separation because of a high loss of protein in the 0.2-µm SWM [129]. Therefore, establishing an optimal process of SWM based on the pore size can increase the separation efficiency of milk components from cell culture media. In summary, the milk components present within cell culture media can be effectively separated into fat globules, casein micelles, and whey proteins by implementing an optimal process that integrates microfiltration, ultrafiltration, and nanofiltration, using SWM.

## Conclusions

Cellular agriculture in the dairy sector may provide a wide range of opportunities for the sustainable production of dairy products, such as milk components or cell-cultured milk. Cellular agriculture in the dairy sector is categorized into fermentation-based and animal cell culture-based cellular agriculture. While several companies predominantly focus on producing milk components through fermentation-based technology, precision fermentation technology still faces the challenges with respect to GMO concerns. Several startup companies are attempting to produce milk components by cultivating ECs in animal cell culture-based technology. However, the technology is still in its early stages of development. This review summarized the structural and functional attributes of the mammary gland and discussed the technologies for cell-cultured milk production. The major technologies were (1) resourcing of mammary EC line, (2) optimizing the cell culture media, (3) establishing the cell culture system, and (4) down-stream processing of milk component. Additionally, future developments and areas of further research include several key areas. Firstly, there is a need to efficiently cultivate milk-derived mammary ECs, achieved through the identification of reliable biomarkers and the use of optimal proliferation and differentiation media. Secondly, the application of 3D cell culture techniques, bioreactors, and membrane separation systems holds promise for scaling up the production of cell-cultured milk. Lastly, it is imperative to reduce the cost of cell-cultured milk compared to traditional milk, ensuring consumer accessibility to the product. In conclusion, this review presents current insights and challenges regarding cell culture-based dairy production and offers implications for ongoing efforts required to produce commercially significant quantities of cell-cultured milk.

## Data Availability

Not applicable.

## Abbreviations

2D: Two-dimensional
3D: Three-dimensional
BLG: β-lactoglobulin
BM: Basement membrane
CK: Cytokeratin
CORT: Cortisol
DMEM/F12: Dulbecco’s Modified Eagle’s Medium/Nutrient Mixture F-12 Ham
E1: Estrone
E2: 17β-Estradiol
ECM: Extracellular matrix
ECs: Epithelial cells
EDTA: Ethylene-diamine-tetraacetic acid
EGF: Epidermal growth factor
FBS: Fetal bovine serum
FBs: Fibroblasts
FCS: Fetal calf serum
GMO: Genetically modified organism
IGF-1: Insulin growth factor-1
INS: Insulin
ITS: Insulin-transferrin-selenium
MCs: Myoepithelial cells
Mt: Million metric tons
MUFA: Monounsaturated fatty acid
P4: Progesterone
PRL: Prolactin
PUFA: Polyunsaturated fatty acid
SFA: Saturated fatty acid
α-SMA: α-Smooth muscle actin
SWM: Spiral-wound membrane
TCM: Tubular ceramic membrane
